# Prokineticin Is a New Linker between Obesity and Cardiovascular Diseases

**DOI:** 10.3389/fcvm.2017.00020

**Published:** 2017-04-12

**Authors:** Canan G. Nebigil

**Affiliations:** ^1^CNRS-University of Strasbourg, UMR 7242, Illkirch, France

**Keywords:** prokineticin, G protein-coupled receptors, obesity, diabetes, anorexic, angiogenic hormones

## Abstract

Obesity is a fast growing epidemic event worldwide. Fatness is associated with a number of comorbidities, including cardiovascular diseases (CVDs). Although obesity can be heredity in 30–70% cases, the environmental contributions also play an important role in the increasing prevalence of obesity. The relationship between development of obesity and CVD is poorly characterized. Obesity and CVD can also be resulted from a common mechanism such as metabolic, inflammatory, and neurohormonal changes. Prokineticins are defined as cytokines (immunoregulatory proteins), adipokines (adipocyte-secreted hormone), angiogenic (increasing vessel formation), or aneroxic (lowering food intake) hormones. Prokineticin-mediated signaling plays a key role in the development of obesity and CVD. Two forms of prokineticins exist in circulation and in various tissues including the brain, heart, kidney, and adipose. Prokineticins act on the two G protein-coupled receptors, namely, PKR1 and PKR2. Prokineticin-2 (PK2) *via* PKR1 receptor controls food intake and prevents adipose tissue expansion. The anti-adipocyte effect of PKR1 signaling is due to suppression of preadipocyte proliferation and differentiation capacity into adipocytes. PK2/PKR1 signaling promotes transcapillary passages of insulin and increases insulin sensitivity. It also plays an important role in the heart and kidney development and functions. Here, we discuss PK2 as a new adipocytokine in the association between obesity and CVD. We also highlight targeting PKR1 can be a new approach to treat obesity and CVD.

## Introduction

Obesity is a major health problem in worldwide regardless of sex and age (1). It is both an independent risk factor and a risk marker for the development of asymptomatic and symptomatic cardiovascular disease (CVD) (2). Common pathways may involve in the pathogenesis of obesity and CVD. Indeed, CVD can occur due to structural and functional changes of the myocardium through excess fat deposition and constant and unremitting metabolic stress related to obesity (2). Interestingly, anti-obesity therapies with anorexic peptides improve cardiovascular function and reduce cardiovascular morbidity and mortality (3). Recent evident showed that some brain regions is involved in food intake regulation and also play an important role in regulation of cardiovascular-blood homeostasis (4). Therefore, it is important to delineate the common mechanisms regulating both obesity and cardiovascular events for development of novel therapeutics. Here, we outlined the current information on the role of anorexic and angiogenic peptide prokineticin signaling in obesity and CV-renal diseases.

## Prokineticins and Their Receptors

Prokineticins are released by monocytes, macrophages, and reproductive organs (5). A high level of prokineticins has been found in obese human AT (6), as well as heart and kidney (7, 8). Two isoforms of prokineticins have been identified: prokineticin-1 and prokineticin-2 (PK2).

## PK2 is an Anorexic Peptide

The regulation of food intake is a complex process involving reciprocal signals between the central nervous system and the periphery. The region in the hypothalamus governing a feeding and energy homeostasis is called as arcuate nucleus (ARC). The ARC contains primary neurons that express neuropeptides with opposing effects on food intake. ARC neurons release anorexic peptide such as the proopiomelanocortin (POMC)-derived peptide, alpha-melanocyte-stimulating hormone, and cocaine, amphetamine-regulated transcript (CART) peptide (9). However, in the ARC, neuropeptide Y (NPY)-producing neurons have been shown to stimulate food intake.

Prokineticin-2 controls food intake and fat tissue expansion through actions in the ARC in the hypothalamus (10). Mainly, PKR1 receptors are expressed in the NPY/AgRP and POMC/CART neurons. Intracranial injection of PK2 in rats abolishes food intake, whereas anti-PK2 antibody increases food intake. Anorexic effect of PK2 is mediated at least partly *via* the hypothalamic ARC melanocortin system. Indeed, PKR1 is the first non-melanocortin G protein-coupled receptors to be regulated by the melanocortin receptor accessory protein 2 that inhibits specifically PKR1 signaling (11).

Peripheral administration of PK2 reduces food intake and body weight in both lean mice and diet-induced obesity models (12). Global ablation of PK2 in mice leads to obesity. Hypothalamic PK2 levels were found extremely high in the early neonatal period. However, a low level of PK2 was observed under fasting conditions (13). The inactivating mutations of PK2 gene and the obesity have been correlated in human (12, 14). Anorexic effect of PK2 was completely absent in the PKR1 deficient mice (12), indicating that the anorexic effects of PK2 are mediated by PKR1 in the hypothalamus.

## Prokineticin in Obesity

Obesity can be resulted from adipocyte hypoplasia/hyperthrophy accompanied with inflammation of AT, defective of extracellular matrix remodeling, fibrosis, and an altered secretion or expression of adipokines (15). PK2 releases from AT in obese individuals; however, it suppresses AT expansion by two distinct mechanisms: the central regulation of food intake and limiting preadipocyte function.

In isolated preadipocytes, PKR1 activation suppresses proliferation and adipogenic differentiation (6). Indeed, an abnormally excessive abdominal fat mass accumulation was observed in adipose tissue-specific PKR1-deficient (PKR1^*ad*−/−^) mice (6). The expansion of AT in both PKR1 null and PKR1^*ad*−/−^ mice was due to formation of new adipocytes. These mice displayed an acceleration of preadipocyte proliferation and differentiation. Despite PKR1*^null^* and PKR1^*ad*−/−^ mice display abdominal obesity, only PKR1*^null^* mice have peripheral obesity with a diabetes-like syndrome (6). Thus, non-adipocyte PKR1-mediated events may contribute to the development of a diabetes-like syndrome. Angiogenesis has important roles in the modulation of insulin sensitivity and expansion of AT (16). Indeed, endothelial-specific PKR1 knockout mice (PKR1^*ec*−/−^) had insulin resistance in adipocytes (17). Insulin cannot promote normal fat storage, resulting in excess circulating free fatty acids that, in turn, further contribute into insulin resistance in muscle, leading to diabetes-like syndrome in PKR1^*ec*−/−^ adipocytes.

The expansion of AT in obesity is also required a shift in the polarized states of macrophages from the M2 to the pro-inflammatory M1 form (18). PK2 promotes inflammatory phenotype of mouse macrophages (19) and reduces IL-10 and IL-4 production in mice splenocytes (20). In contrast, PKR1^*ad*−/−^ mice displayed substantial infiltration of macrophage in the AT. Whether PKR1 signaling retains an M2 polarization, or triggers the phenotypic switch from M1 to M2 to preserve adequate adipocyte function in obesity is unknown.

## Prokineticin in Insulin Resistance

The transcapillary delivery of insulin from endothelial cells (ECs) to the skeletal muscle is the rate-limiting step in insulin-stimulated glucose uptake (21). The defect in insulin delivery process *via* ECs contributes to insulin resistance (22). Thus, the vascular endothelium is considered as a potential therapeutic target for prevention of insulin resistance and related complications (23).

Endothelial cell-specific PKR1 knockout (PKR1^ec−/−^) mice exhibited impaired capillary formation and low transcapillary insulin uptake, which was rescued by PKR1 gene transfection by adenovirus (17). Overexpressing PKR1 in EC promotes insulin transendothelial uptake (24) and angiogenesis (25). These data highlight the role of PKR1 as a positive regulator of insulin uptake (26). In concert with this *in vitro* finding, PKR1^*ec*−/−^ mice exhibit hyperphagia and severe lipodystrophy due to poor capillary formation in the AT. Lipodystrophies, involving a loss of AT, are known to induce hyperphagia and peripheral insulin resistance (27). Impaired insulin delivery and signaling in ECs have also been observed in human patients with type 2 diabetes and obesity with insulin resistance (28).

Therapeutic strategies targeting PKR1 could be important to treat obesity and obesity-associated insulin resistance, since PKR1 signaling suppresses appetite, reduces adipocyte expansion, promotes normal fat storage, and increases insulin sensitivity.

## Prokineticin in Heart Development and Function

PKR1 regulates epicardial–mesenchymal transition to form epicardial-derived progenitor cell (EPDC) during cardiogenesis (29). Genetic ablation of PKR1 in epicardium (PKR1^*wt1*−/−^) leads to a ventricular hypoplasia, septal defects, and deficient vascularization, leading to embryonic lethality. Epicardial PKR1 contributes to cardiomyocyte proliferation in a paracrine pathway that is required for the development of ventricular wall. Epicardial PKR1 is also a key signaling for EPDC proliferation and differentiation into vasculogenic cell type, involved in formation of coronary circulation (30).

Altered expression of prokineticins and their receptors has been implicated in heart failure (31) and aortic rupture (32). Prokineticin signaling plays an important role especially in cardiac progenitor cell commitment and cell-to-cell communications (30).

PKR1 signaling protects cardiomyocytes against hypoxia-mediated apoptosis by activating Akt signaling pathway (7). Transgenic mice-overexpressing PKR1 in the cardiomyocytes exhibits an increased number of EPDCs, associated with increased number of vessels (30). Indeed, the cardiac PKR1 signaling up-regulates its own ligand PK2 to stimulate the EPDC differentiation into endothelial and smooth muscle cells to promote neovasculogenesis (30). Interestingly, PKR1^null^ mice displayed cardiomyocyte contractile defects and apoptosis partially due to lack of PKR1 signaling in cardiomyocytes (8). These data indicate that cardiomyocyte PKR1 is essential for cardiomyocyte survival and contractility with a cell autonomous way. However, cardiomyocyte PKR1 derives EPDCs proliferation and differentiation.

In cardiac ECs, PKR1 activates Akt and MAPK to promote proliferation, migration, and angiogenesis (25). Accordingly, loss of PKR1 in ECs leads to defective angiogenesis (17). The posterior walls of PKR1^ec−/−^ hearts were thinner due to the loss of capillary formation and a high level of apoptosis (17). Indeed, PKR1^ec−/−^ hearts displayed ectopic lipid deposition and abnormal insulin signaling together with capillary defects, resulting in impaired diastolic function. Abnormal insulin signaling was due to defective transcapillary transport of insulin in the vascular wall of PKR1^ec−/−^mice. In accord with this *in vivo* findings, isolated ECs from the mutant cardiac and renal tissues exhibited very little insulin uptake, confirming that the loss of PKR1 from ECs decreased insulin transport (17). Indeed, activation of PKR1 in ECs promoted FITC-insulin passage. Nitric oxide deficiency in the ECs is associated with the insulin resistance and endothelial dysfunction (33). Similarly, in the endothelium of patients with diabetes mellitus, insulin-mediated eNOS activation is altered (34). In agreement, insulin uptake and insulin-mediated eNOS activation were impaired in PKR1-deficient ECs. Impaired transcapillary insulin delivery leads to defective eNOS activation, affecting endothelium-dependent relaxation in PKR1^ec−/−^aortas (17). These impairments in PKR1^ec−/−^ mice resulted in hypertension at the later age. These mice models should facilitate studies of both pathogenesis and therapy of cardiac disorders in humans.

## Prokineticin in Renal Development and Function

In contrast to developing heart, PKR1 is necessary for renal mesenchymal–epithelial transition (MET) that is involved in formation of renal progenitors, regulating glomerulogenesis toward forming nephrons during kidney development (29). Indeed, PKR1 activates NFATc3 and modifies MET processing involved in the development of nephron. Mutant mice with targeted PKR1 gene disruptions in nephron progenitors has been shown to exhibit partial embryonic and postnatal lethality due to hypoplastic kidneys with premature glomeruli and necrotic nephrons. Kidney developmental defects in these mice were manifested in the adult stage as renal atrophy with glomerular defects, nephropathy, and uremia (29).

PKR1 knockout mice also displayed renal tubular dilation, reduced glomerular capillaries, urinary phosphate excretion, and proteinuria (8). Similarly, PKR1^ec−/−^ mice displayed enlarged tubular structures with a swollen necrotic nucleus, abnormal mitochondria, and aberrant organization of podocytes, dilatation of the Bowman’s spaces in the glomeruli, a compact glomerulus, and fibrosis. Defects in tubular and glomerular structures are associated with functional abnormalities such as high levels of creatinine clearance and proteinuria (8). Indeed, increased excretion of absolute renal phosphate (Pi) in the PKR1^ec−/−^ mice is due to lower levels of sodium–calcium and sodium–phosphate exchanger. The morphological changes in the PKR1^ec−/−^ kidneys were accompanied with apoptosis impaired insulin signaling and lipid accumulation. Endothelial dysfunction resulted from loss of PKR1 signaling underlies the pathological features of heart and kidney.

## Conclusion

**Figure 1:**
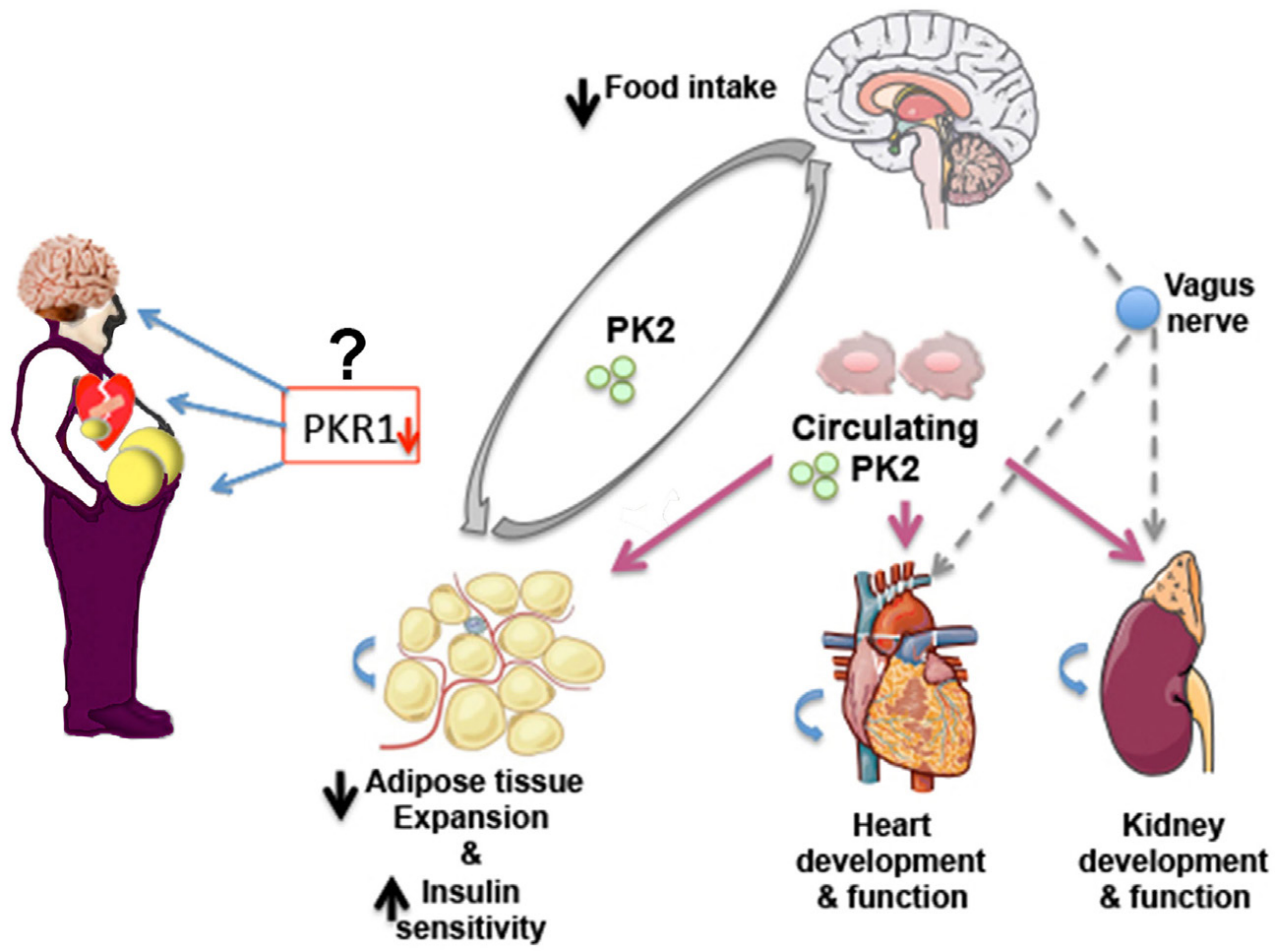
**Prokineticin-2 (PK2)/PKR1 signaling may act as a new connector between development of obesity, diabetes and cardiovascular diseases (CVDs)**. PKR1 deficiency promotes WAT expansion and insulin resistance, CVD, and alters food intake in mice. Whether reduced level of PKR1 or functional mutated PKR1 involves these disorders in human needs to be studied. PK2/PKR1 signaling in central nervous system (CNS) regulates food intake. PK2 released from adipocytes controls preadipocyte conversion to adipocyte *via* PKR1 signaling and may affect food intake *via* CNS. Circulating or local PK2 signaling *via* PKR1 contributes development and function of heart and kidney. Whether this signaling involves heart and kidney regulation *via* CNS remains to be study.

Multiple biological mechanisms linking obesity and CVD events have been identified. Identification of signaling pathways linking obesity and CVD is important for development of novel therapeutics. PKR1 signaling plays an important role in central regulation of appetite, the suppression of adipocyte mass and insulin sensitizing effects on skeletal muscle and other tissues, cardiac regeneration, and kidney development and function (Figure 1). Whether PKR1 signaling regulates heart and kidney function *via* vagus nerve remains to be study. Recently, PKR1 non-peptide agonist has been discovered (35), which it prevents cardiac lesion formation and improves cardiac function after myocardial infarction in mice, promoting proliferation of cardiac progenitor cells and neovasculogenesis. Targeting PKR1 can be a novel therapeutic approach to treat obesity, diabetes and CVD.

## Author Contributions

The author confirms being the sole contributor of this work and approved it for publication.

## Conflict of Interest Statement

The author declares that the research was conducted in the absence of any commercial or financial relationships that could be construed as a potential conflict of interest.
